# Aberrant RNA m^6^A modification in gastrointestinal malignancies: versatile regulators of cancer hallmarks and novel therapeutic opportunities

**DOI:** 10.1038/s41419-023-05736-w

**Published:** 2023-04-04

**Authors:** Li-Ting Shen, Lin-Rong Che, Zongsheng He, Qian Lu, Dong-Feng Chen, Zhong-yi Qin, Bin Wang

**Affiliations:** 1 Department of Gastroenterology & Chongqing Key Laboratory of Digestive Malignancies, Daping Hospital, Army Medical University (Third Military Medical University), Chongqing, 400042 China; 2 Department of Internal Medicine, Hospital of Zhejiang Armed Police (PAP), Hangzhou, 310051 China; 3 Institute of Pathology and Southwest Cancer Center, and Key Laboratory of Tumor Immunopathology of Ministry of Education of China, Southwest Hospital, Army Medical University (Third Military Medical University), Chongqing, 400038 China; 4 Jinfeng Laboratory, Chongqing, 401329 China

**Keywords:** Cancer epigenetics, Gastrointestinal cancer

## Abstract

Gastrointestinal (GI) cancer is one of the most common malignancies, and a leading cause of cancer-related death worldwide. However, molecular targeted therapies are still lacking, leading to poor treatment efficacies. As an important layer of epigenetic regulation, RNA N6-Methyladenosine (m^6^A) modification is recently linked to various biological hallmarks of cancer by orchestrating RNA metabolism, including RNA splicing, export, translation, and decay, which is partially involved in a novel biological process termed phase separation. Through these regulatory mechanisms, m^6^A dictates gene expression in a dynamic and reversible manner and may play oncogenic, tumor suppressive or context-dependent roles in GI tumorigenesis. Therefore, regulators and effectors of m^6^A, as well as their modified substrates, represent a novel class of molecular targets for cancer treatments. In this review, we comprehensively summarize recent advances in this field and highlight research findings that documented key roles of RNA m^6^A modification in governing hallmarks of GI cancers. From a historical perspective, milestone findings in m^6^A machinery are integrated with a timeline of developing m^6^A targeting compounds. These available chemical compounds, as well as other approaches that target core components of the RNA m^6^A pathway hold promises for clinical translational to treat human GI cancers. Further investigation on several outstanding issues, e.g. how oncogenic insults may disrupt m^6^A homeostasis, and how m^6^A modification impacts on the tumor microenvironment, may dissect novel mechanisms underlying human tumorigenesis and identifies next-generation anti-cancer therapeutics.

**Figure:**
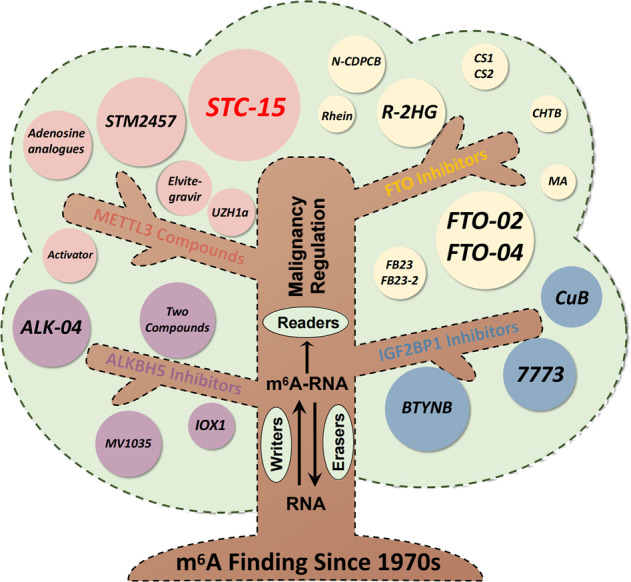
In this review, we discuss advances in our understanding of m^6^A RNA modification since its discovery in the 1970s to the latest progress in defining its potential clinic relevance. We summarize the molecular basis and roles of m^6^A regulators in the hallmarks of GI cancer and discuss their context-dependent functions. Furthermore, the identification and characterization of inhibitors or activators of m^6^A regulators and their potential anti-cancer effects are discussed. With the rapid growth in this field there is significant potential for developing m^6^A targeted therapy in GI cancers.

In this review, we discuss advances in our understanding of m^6^A RNA modification since its discovery in the 1970s to the latest progress in defining its potential clinic relevance. We summarize the molecular basis and roles of m^6^A regulators in the hallmarks of GI cancer and discuss their context-dependent functions. Furthermore, the identification and characterization of inhibitors or activators of m^6^A regulators and their potential anti-cancer effects are discussed. With the rapid growth in this field there is significant potential for developing m^6^A targeted therapy in GI cancers.

## Facts

- m^6^A, the most abundant epigenetic modification on RNA, regulates both RNA metabolism and chromatin accessibility.
- Dysregulation or mutation of m^6^A machinery drives tumorigenesis in GI cancers.
- The context-dependent functions of m^6^A regulators are observed in different cancer types as well as cancers with distinct genomic alterations.

## Open Questions

- How does m^6^A machinery orchestrate the hallmarks of cancer in different genetic backgrounds?
- How does oncogenic stimuli act on m^6^A machinery to regulate tumor initiation and progression?
- What are novel m^6^A-independent functions for these m^6^A regulators?
- How can dysregulated m^6^A machinery be precisely targeted to developed novel anti-cancer strategies for clinical translation?

## Introduction

The deposition of chemical modifications onto RNA is an efficient way of regulating gene expression in a temporal and spatial manner [1]. To date, more than 170 types of RNA modifications have been identified [2]. Among these modifications, methylation of N6 adenosine to produce N6-methyladenosine (m^6^A) is the most common type of RNA modification, which happens predominantly at the consensus RRACH motif (where R represents G or A, H represents A, C or U, and A represents the m^6^A modified adenosine) which is often found within the 5′ or 3′ untranslated regions (UTRs) of mRNAs [3, 4]. m^6^A is now considered a key modification that regulates gene expression by influencing fundamental RNA-related processes including RNA stability, alterative splicing, nuclear export, translation, and decay, as well as biological processes including phase separation [5–10]. Technological advances in characterizing m^6^A have been made through the recent development of m^6^A immunoprecipitation and sequencing techniques, which has led to the discovery of enzymes that regulate m^6^A. These enzymes include m^6^A methyltransferases (writers), m^6^A demethylases (erasers) and m^6^A-binding proteins (readers), which regulate m^6^A modification in a dynamic and reversible manner. Aberrant m^6^A methylation caused by germline mutations, or altered expression of these modulators, has been found to influence numerous tumorigenic properties including proliferation, progression, and immunomodulatory abnormality [11, 12].

Gastrointestinal (GI) malignancy is one of the major causes of cancer-related death worldwide with limited options of molecular targeted therapies. In light of tremendous progresses elucidating the roles of m^6^A modification in regulating tumorigenesis, aberrant m^6^A methylation may serve as a promising molecular target for therapeutic intervention of GI cancers. With no comprehensive review to date on the roles of m^6^A in regulating GI cancers and few therapeutic strategies available, it is timely to summarize how m^6^A modification dictates various malignant hallmarks of GI cancers. Given increasing reports of small chemical compounds targeting the m^6^A regulatory machinery, a comprehensive review is also warranted to aid translational studies targeting RNA m^6^A regulatory pathways for anti-cancer therapeutics. Therefore, we summarize recent advances in the biological functions and underlying molecular mechanisms of m^6^A modification in GI cancer and the prospects for targeting m^6^A as a new therapeutic strategy in human cancers.

## The machinery of the RNA m^6^A modification pathway

RNA m^6^A is elaborately controlled by proteins involved in writing, erasing, and reading this modification. Elucidating the detailed mechanisms of these m^6^A regulators may provide cues for the development of targeted therapies for GI cancers.

### Writers

The canonical m^6^A methyltransferase complex (MTC) is comprised of methyltransferase-like 3 (METTL3), METTL14, and Wilms’ tumor 1-associating protein (WTAP), which largely determines whether the downstream RNA should be modified by m^6^A methylation. METTL3 is the indispensable catalytic enzyme that transfers the methyl group from S-Adenosylmethionine (SAM) to N6 adenine [13]. This process is precisely coordinated with Exon Junction Complexes (EJCs) to provide specificity of m^6^A deposition [14]. METTL3-mediated m^6^A modification on chromatin-associated RNA promotes the formation of DNA:RNA hybrids, which have been widely found in many activated regulatory chromosomal loci and G-rich sequences such as telomeres and centromeres [15]. More importantly, modifying RNA through m^6^A can regulate genome stabilization and integrity by modulating the RNA component of DNA:RNA hybrids [16], suggesting that RNA modifications contribute to genome maintenance. METTL14, another key enzyme of the MTC, contains a SAM-binding motif and forms stable heterodimer complexes with METTL3 [17], which play an essential role in m^6^A deposition on nuclear RNAs by enhancing catalytic efficacy [9, 18]. Furthermore, deletion of METTL14 inhibits self-renewal and differentiation abilities of embryonic stem cells [19]. While WTAP, another MTC core factor, has no direct catalytic activity for m^6^A modification [20], it works as an adaptor protein that binds both METTL3 and METTL14, thus stimulating accumulation of nuclear speckles, a crucial process in m^6^A deposition [18]. In addition, WTAP has been found to interact with other regulatory proteins such as ZC3H13 and KIAA1429, and long non-coding RNAs (lncRNAs), suggesting that WTAP may serve as a molecular scaffold to recruit other factors to cooperate with the MTC [21].

Recently, METTL16 has also been identified as a methyltransferase for the U6 spliceosomal small nuclear RNA, thereby regulating the stability and splicing of mRNAs [22–24]. Furthermore, METTL5 has been identified as an m^6^A methyltransferase, catalyzing m^6^A on structured RNAs including 18S rRNA and 28S rRNA independent of the MTC [25]. METTL5 can form a heterodimeric complex with TRNA Methyltransferase Activator Subunit 11-2 (TRMT112) to enhance its own metabolic stability in cells. The crystal structure of the METTL5-TRMT112 complex reveals that the RNA-binding module of the complex differs largely from that of other m^6^A RNA methyltransferases [25]. In addition, several other m^6^A writers such as ZC3H13 and KIAA1429 have been reported to achieve precise post-transcriptional modulation by selectively recognizing candidate methylation sites or docking MTC to nuclear speckles [26–28].

Although a series of novel components have been progressively identified and characterized, how the MTC specifically localizes to transcriptional products remains largely unknown. There have been two hypotheses proposed to address this; 1) the MTC may interact with transcription factors (TFs) thus catalyzing the m^6^A modification on transcripts in a TF-dependent manner [29], and 2) specific histone modifications could recruit MTC components to transcripts, such as histone H3K36me3 being shown to directly guide METTL14 localization to the genome in HepG2 cells [30].

### Erasers

m^6^A eraser proteins remove m^6^A modifications by recruiting ferrous iron as a co-factor and α-ketoglutarate as a co-substrate [31]. Unlike the numerous types of m^6^A methyltransferases, only two m^6^A demethylases have been identified, the fat mass and obesity-associated (FTO) and the AlkB homolog 5 (ALKBH5) proteins. FTO, the first identified m^6^A eraser protein, which eliminates m^6^A by demethylating both internal m^6^A and N6, 2ʹ-O-dimethyladenosine (5ʹ cap m^6^A) mRNA [32, 33], preferentially binds to pre-mRNAs in intronic regions and regulates alternative splicing and 3ʹ end processing [7]. In addition, FTO mRNA demethylation can induce the translation of mRNAs into proteins such as shown with the GAP43 mRNA [34]. More recently, FTO was found to remove m^6^A from chromosome-associated regulatory RNAs (carRNAs) including Long-Interspersed Element-1 (LINE1), particularly in mouse embryonic stem cells (mESCs) to ensure mouse oocyte and embryonic development, indicating a crucial role for m^6^A in chromosome state shaping. The discovery of FTO was a milestone finding as it provided evidence that the process of m^6^A methylation is dynamic and reversible [35, 36].

The second eraser protein is ALKBH5, shows greater specificity in m^6^A demethylation compared with FTO. Its demethylation activity influences mRNA export and RNA metabolism by reducing the level of m^6^A in nuclear speckles [37]. In ALKBH5-deficient cells, cytoplasmic RNA levels are substantially increased due to accelerated export of nuclear RNAs and upregulation of nascent RNAs [37]. ALKBH5-deficient male mice are infertile due to the abnormal apoptosis of spermatocytes. Furthermore, ALKBH5 deletion increases exon skipping and causes rapid degradation of aberrantly spliced transcripts [38]. These studies indicate that reversible m^6^A of RNA has fundamental and broad functions in cells.

### Readers

The interrelationship between m^6^A writers and erasers determines the dynamic and reversible regulation of m^6^A modifications. The m^6^A modification exerts is biological consequence through the binding m^6^A readers such as the YT521-B homology (YTH) domain-containing proteins (YTHDFs), insulin-like growth factor 2 mRNA binding proteins (IGF2BPs), and heterogeneous nuclear ribonucleoproteins (HNRNPs). YTHDF proteins contain a C-terminal YTH domain important for their binding to the m^6^A modified mRNA while the N-terminal region is flexible and serves as a regulatory region to bind various co-factors [39]. More specifically, YTHDF1 selectively recognizes m^6^A-modified mRNAs through the YTH domain and interacts with initiation factors to improve their translation via the N-terminal domain [6]. YTHDF1 also binds to m^6^A localized in the coding sequence of certain mRNAs to regulate translation elongation [40]. Conversely, YTHDF2 selectively binds m^6^A-modified RNA and accelerates the decay of m^6^A-modified transcripts by recruiting the CCR4-NOT complex [41, 42]. Interestingly, YTHDF3 works with YTHDF1 to synergistically enhance translation of methylated RNAs but also accelerates mRNA decay by interacting with YTHDF2 [42, 43]. Therefore, YTHDF3 may function to mediate RNA accessibility of YTHDF1 and YTHDF2.

IGF2BPs, including IGF2BP1-3, also function as a distinct family of m^6^A readers. IGF2BPs structurally contain six canonical RNA-binding domains, two RNA recognition motifs (RRM), and four K homology domains [44]. IGF2BPs recognize the m^6^A target sequence via the K homology domains and promote mRNA stability by binding to target transcripts through GG(m^6^A)C, a typical m^6^A consensus [45]. In contrast to the mRNA-decay-promoting function of YTHDF2, IGF2BPs binding to m^6^A enhances the stability and translation efficiency of their targeted mRNAs [45]. In addition, IGF2BPs binding sites on RNA show limited overlap with YTHDF2 which, at least in part, explain why IGF2BPs play an opposing role in their regulation of m^6^A-modified mRNA fate [45].

Among the hnRNP family, hnRNP-A2B1 was the first identified as a m^6^A binding protein that enhances the processing of primary miRNAs (pri-miRNAs) by interacting with RNA-binding protein DGCR8 in an m^6^A-dependent manner [46]. hnRNP-A2B1 is also involved in modulating alternative splicing of mRNA transcripts [46, 47]. Similarly, hnRNP-C and hnRNP-G regulate mRNA abundance and splicing by processing m^6^A-modified RNA transcripts [48, 49]. The m^6^A site of hnRNA indirectly alters the binding of hnRNP-C/G to its U-tract motifs, which facilitates the binding of transcripts to hnRNP-C and hnRNP-G and thereby modulates mRNA abundance and splicing. This phenomenon is termed a “m^6^A-switch”.

## Multifaceted roles of m^6^A modification in orchestrating mRNA fate

**Fig. 1:**
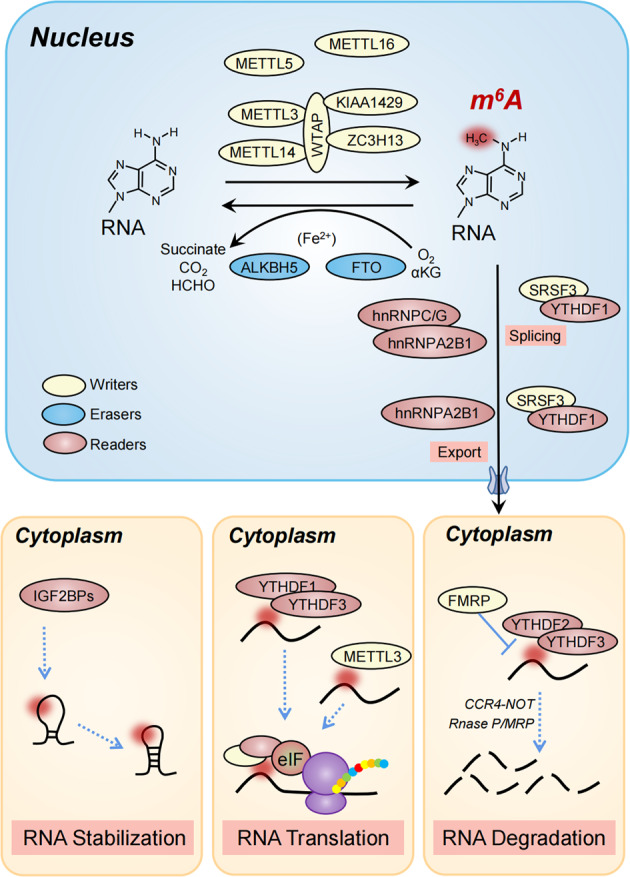
The writers, erasers and readers in the reversible m^6^A modification. m^6^A RNA modifications are catalyzed by the multicomponent methyltransferases complex (MTC), including the core factors composed by METTL3, METTL14, and WTAP and additional co-factors such as METLL5 and METLL16. FTO and ALKBH5 trigger the removal process by recruiting ferrous iron as a co-factor and α-ketoglutarate as a co-substrate. m^6^A modified RNAs are recognized by reader proteins such as hnRNPs to facilitate splicing and nuclear export of mRNAs. Cytoplastic m^6^A modified RNAs identified by IGF2BPs and YTH domain containing proteins contribute to stabilization, enhanced translation, and degradation in reader-dependent processes.

With the development of advanced sequencing technology, the resolution of m^6^A detection has evolved from determining bulk m^6^A:A ratios, to being capable of identifying precisely modified adenines in the whole transcriptome, which has led to a series of online databases compiling the vast amount of sequencing data (Table 1 [3, 4, 33, 50–63]). These methodological advancements have pushed forward our understanding of the role of m^6^A in regulating mRNA metabolism. Due to the wide range of m^6^A modifications in the transcriptome, m^6^A has been shown to influence every stage of mRNA processing including splicing, nuclear export, translation, and decay (Fig. 1).

**Table 1:** Methodologies for m^6^A detection.

Bulk quantitative analysis	Dot blot, LC-MS/MS [49]	
Precise detection and quantification	m^6^A specific antibody-dependent	MeRIP-seq^3^ (m^6^A-seq^4^), miCLIP-seq [33], m6A-CLIP [52]; m^6^ACE-seq [53]
	m^6^A specific antibody-independent	SCARLET [54], MAZTER-seq [55], m^6^A-REF-seq [56], DART-seq [57], NOseq [58]
Prediction methods and web servers	WHISTLE [59]: http://whistle-epitranscriptome.com.	
	RADAR [60]: https://github.com/scottzijiezhang/RADAR.	
	m6AVAR [61]: http://m6avar.renlab.org	
	RMBasev2.0 [62]: http://rna.sysu.edu.cn/rmbase/	
	MoAIMS [63]: https://github.com/rreybeyb/MoAIMS	
	m^6^A-Atlas [64]: www.xjtlu.edu.cn/biologicalsciences/atlas

### Regulation of mRNA splicing

Multiple studies have revealed that dynamic m^6^A modifications alter mRNA splicing. In particular, mRNAs undergoing alternative splicing contain greater levels of m^6^A modifications than transcripts undergoing single isoform splicing [4, 64]. This role for m^6^A in mRNA splicing has been further demonstrated in *Drosophila*, where mutation of IME4 (a METTL3 homolog) effects female-specific splicing of the Sex-lethal gene [26, 65]. In mammals, deletion of METTL3 facilitates intron retention mouse embryonic stem cells and RNA splicing in pancreatic cancer [19, 66]. In addition, WTAP is enriched in protein complexes modulating pre-mRNA splicing [21], further suggesting a role for m^6^A in mRNA splicing. In addition to m^6^A writer proteins, dysregulation of m^6^A eraser proteins also impacts alternative splicing patterns [38]. For instance, FTO modulates mouse pre-adipocyte differentiation by regulating alternative splicing of adipogenesis-related mRNAs [67]. Altered RNA splicing is also observed in ALKBH5 deficient cells [37]. While m^6^A writers and erasers regulate alternative splicing by influencing the direct levels of m^6^A modified RNA, m^6^A reader proteins can also directly regulate splicing machinery [49, 68]. For instance, m^6^A-bound YTHDC1 related splicing factor SRSF3 has been shown to block binding of SRSF10 to m^6^A-modified RNA, which promotes exon inclusion [69]. Similarly, hnRNPA2B1 has also been implicated in the regulation of alternative splicing [46]. Taken together, these studies confirm an important role for m^6^A modification in mRNA splicing.

### Regulation of mRNA export

Export of mRNAs from the nucleus is also enhanced by m^6^A modification. For instance, depletion of the m^6^A writer METTL3 suppresses mRNA export [70]. Similarly, depletion of the m^6^A eraser ALKBH5, increases levels of cytoplasmic m^6^A-containing mRNAs due to accelerated nuclear RNA export [37]. Also, the reader protein YTHDC1 facilitates the export of m^6^A-modified transcripts by binding to nuclear transporters. For instance, the m^6^A methyltransferase complex recruits the TREX mRNA export complex to m^6^A-modified mRNAs, which stimulates the recruitment of YTHDC1 and downstream nuclear transport receptors [71]. Another study showed that YTHDC1 enhances the export of m^6^A-modified mRNAs by interacting with nuclear RNA export factor 1 [8]. Another reader, hnRNPA2B1, enhances m^6^A modified transcripts to undergo nucleocytoplasmic trafficking as well [72]. These studies indicate that the m^6^A modification of mRNAs accelerates their nuclear export.

### Regulation of mRNA translation

m^6^A can influence mRNA translation via various mechanisms. Several reader proteins including YTHDF1 and YTHDF3 have been reported to promote translation of target mRNAs. YTHDF1 selectively binds to m^6^A sites around the stop codon and collaborates with translation initiation factors to enhance the efficiency of cap-dependent translation [6]. YTHDF3 also cooperates with YTHDF1 in the modulation of translation by interacting with ribosomal proteins [43]. Furthermore, eukaryotic translation initiation factor 3 (eIF3) is also reported to directly bind m^6^A in the 5′ untranslated region of transcripts, which recruits the 43S ribosomal complex to facilitate cap-independent translation [73]. The m^6^A writer protein METTL3 promotes translation of a large subset of mRNAs by directly recruiting eIF3 subunit H to the 5′ end [74]. During cellular stress responses, signaling pathways regulating 5′-end cap independent translation are also regulated by m^6^A modification [75]. For instance, nuclear YTHDF2 can bind to the 5′UTR of target transcripts to promote cap-independent translation initiation following heat shock [75].

### Regulation of mRNA decay

It is well documented that m^6^A reader proteins fine-tune gene expression in part by regulating mRNA stability. Among the abovementioned m^6^A reader proteins, YTHDF2 has been reported to be involved in the degradation of m^6^A-modified mRNA through either endoribonucleolytic decay or the exoribonucleolytic cleavage pathway [76, 77]. For instance, heat-responsive protein 12 combines m^6^A-bound YTHDF2 with an endoribonuclease, RNase P/MRP, initiating the endoribonucleolytic cleavage of an m^6^A-containing mRNA [76]. YTHDF2 specifically accesses m^6^A sites and regulates decay of the target transcripts by recruiting the CCR4-NOT deadenylase complex, which promotes mRNA decay via recruitment of exosomes (3′-to-5′ exoribonuclease complex) [41]. In addition, YTHDF2 can accelerate mRNA decay through cooperatively interacting with YTHDF3 or even together with YTHDF1 [6, 78], emphasizing the dual regulatory functions of YTHDF3 in m^6^A-modified mRNA fate decisions. Besides these classic reader proteins, a recent report revealed that fragile X mental retardation protein, a selective RNA-binding protein associated with translating polyribosomes, competes with YTHDF2 for binding to m^6^A-modified mRNAs to block mRNAs from being degraded by YTHDF2 [79, 80].

### Regulation of phase separation

Liquid-liquid phase separation (LLPS), has been recently characterized and considered to play a fundamental role in numerous cellular process such as heterochromatin formation, gene expression, and transcriptomic changes, by defining membraneless liquid compartments such as stress granules (SGs) and processing (P) bodies in the cytosol, or nucleoli, Cajal bodies, and nuclear speckles in the nucleus, consisting of mRNAs and proteins [81, 82]. LLPS requires multivalent interactions, which are mediated by scaffold molecules or proteins with intrinsically disordered regions (IDRs) [83, 84].

Emerging studies suggest that m^6^A modified RNAs, as well as their regulators, play important roles in the formation of LLPS to regulate the fate of mRNAs. The pioneering study in this area uncovered that IDR-contained YTHDFs (Readers) interacts with polymethylated (m^6^A) mRNAs organized scaffolds to comprise various endogenous phase-separated compartments matching with specific molecular functions, including but not limited to the decreased stability and translation efficiency of m^6^A-mRNAs [85]. However, YTHDFs regulated PS formation not only depends on m^6^A mRNA, but also the PS core protein G3BP1 [86]. In stress granules (SGs), deletion of YTHDF1/3 inhibits the localization of both m^6^A mRNA and non-methylated mRNA. Interestingly, YTHDFs-m^6^A-mRNA condensate and G3BP1-mRNA condensate separate from each other regularly, even in the same LLPS structure. Specifically, YTHDF clusters tends to residue on the periphery of G3BP1 clusters which suggests YTHDF clusters may determine the fate of mRNA in a flexible manner by aggregating or disaggregating from granules [87].

Similarly, YTHDC1 containing IDRs can also form LLPS with either m^6^A-mRNAs or enhancer RNAs, leading to mRNA stabilization by blocking interactions with PAXT (polyA tail exosome targeting complex) regulated degradation, or the regulating enhancer and transcription activation, respectively [88, 89]. More Recently, the core catalytic unit METTL3 is capable of driving LLPS condensation partially by self-interaction. Besides, as depositors of m^6^A (the basic writing system), interactions between METTL3 and the other two components, METLL14 or WTAP, may be dependent on the nature of the METLL3 role in forming LLPS indicating that m^6^A writer complex itself is influenced by LLPS as well [90].

## Versatile roles of m^6^A modification in regulating hallmarks of GI cancers

**Fig. 2:**
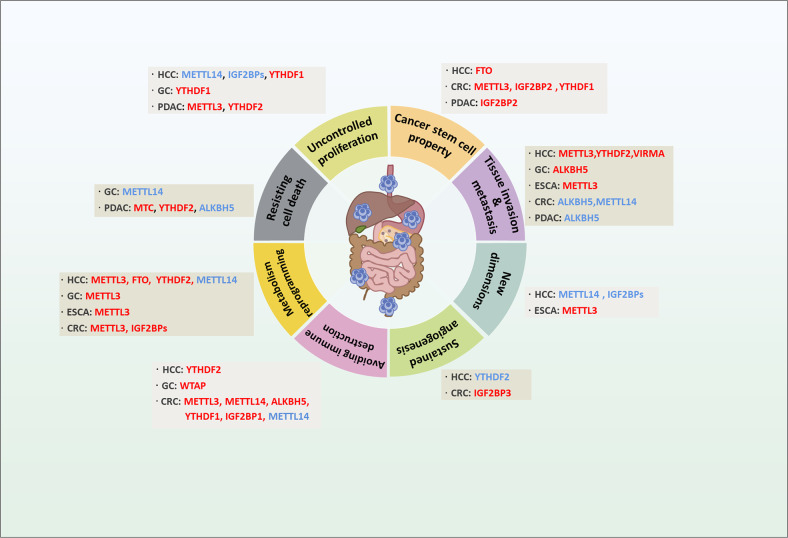
Dysregulation of m^6^A regulators in the hallmarks of GI cancer. Regulators in red represent oncogenic roles in regulating hallmarks of GI and related cancers, and those in blue indicate tumor-suppressive roles. CRC colorectal cancer, ESCA esophageal carcinoma, GC gastric cancer, HCC hepatocellular cancer, PDAC pancreatic ductal adenocarcinoma.

Given the critical roles of m^6^A in regulating mRNA metabolism, it is reasonable to speculate that m^6^A may influence cancer characteristics ranging from sustaining proliferative signaling to distal dissemination. However, the molecular details about how m^6^A influences cellular phenotypes of cancer are still being investigated. Here, we highlight recent insights into the biological functions of m^6^A modification in relation to the hallmarks of GI cancers (Fig. 2).

### Uncontrolled proliferation

Cancer cells sustain chronic proliferation through activation of proliferative and/or pro-survival signals as well as repressing gatekeepers of the cell cycle. The level of m^6^A modification is indirectly correlated with the activity of oncogenic signaling and tumorigenic properties [91]. Suppression of m^6^A via METTL14 depletion promoted gastric cancer (GC) cell proliferation through activating Wnt and PI3K-AKT signaling pathways, while upregulation of m^6^A due to FTO depletion reversed these phenotypical and molecular signaling changes [91]. Similarly, METTL14 also decreases the m^6^A modification on cirORC5 to stimulate its expression, which activates the miR-30C-2-3P/AKT1S1 axis to inhibit GC growth [92]. In Hepatocellular Carcinoma (HCC), In PTEN-deficient pancreatic ductal adenocarcinoma (PDAC), aberrant MTC and YTHDF2 regulation increases PIK3CB mRNA stability through reducing m^6^A levels, which facilitates PTEN-deficient PDAC cell proliferation both in vitro and in vivo via the activation of AKT signaling pathway [93]. Similarly, suppression of the MTC in endometrial cancer due to either METTL14 mutation or reduced expression of METTL3 causes PHLPP2 depletion and mTORC2 accumulation, which increases the activity of AKT signaling pathway [94]. These studies suggest that m^6^A modifiers may serve as potential targets to block AKT signaling and tumor proliferation in cancers. In addition to the AKT pathway, METTL3-induced m^6^A modification of tumor necrosis factor 1 (TNFR1) enhances its expression, contributing to MAPK and NF-κB activation and proliferation of esophageal carcinoma (ESCA). Furthermore, the greater levels of m^6^A modified TNFR1 mRNA indicates the unfavorable outcome of ESCA patients [95].

Bypassing cell cycle arrest is a key characteristic for the malignant proliferation of cancer cells. Recently, PER1, a vital regulator of the mitotic cyclin B1/CDK1 complex [96], is regulated by ALKBH5 in a m^6^A-YTHDF2-dependent manner. As an m^6^A eraser, ALKBH5 depletion increases m^6^A on PER1 mRNA, resulting in cell proliferation in pancreatic cancer, due to the decreased expression of PER1 [97]. In another study, m^6^A modification of cyclin D1 mRNA, the key regulator for G1 phase progression, is regulated in a cell cycle dependent manner. In addition to ALKBH5, FTO demethylates m^6^A modified cyclin D1, and thereby accelerates the degradation of cyclin D1 mRNA, leading to the impairment of the G1-S phase transition [98].

### Resisting cell death

Apoptosis, or programmed cell death, is as a natural process that functions to suppress unchecked cell proliferation. However, tumor cells overcome this restriction and evade apoptosis and other cell death processes including ferroptosis, which are also tightly controlled by m^6^A system. For example, Bcl-2 and Bax, two distinct regulators of apoptosis, are controlled by m^6^A modification [99–101], suggesting that dysregulation of the m^6^A modification to RNA likely contributes to the skirting of apoptosis by cancer cells. METTL14 and IGF2BPs are involved in the stabilization of m^6^A-modified HNF3γ mRNA, which promotes cell apoptosis, thereby sensitizing HCC cells to sorafenib treatment [102]. In pancreatic cancer (PC) cells, depletion of METTL3 decreases the expression of LINC00857, a long non-coding RNA, which promotes apoptosis in PC cells [103]. In addition, YTHDF1 suppresses GC cell apoptosis by promoting USP14 protein translation in an m^6^A-dependent manner [104]. On the other hand, YTHDF1 is upregulated by hypoxia-inducible factor-1α (HIF-1α) in HCC, thus promoting the formation of autophagosomes by enhancing the translation of a series of autophagy-related genes such as ATG2A and ATG14 [104], a role which has been confirmed by YTHDF1 conditional knockout transgenic mice. In PDAC, YTHDF2 facilitates NUCB1 mRNA decay in response to METTL3-mediated m^6^A modification, which leads to autophagy activation and gives rise to the gemcitabine resistance [105]. Suppression of ferroptosis, a novel iron-dependent form of cell death, was shown to be essential for cancer malignant behavior [106, 107], and m^6^A regulators such as METTL14, FTO, YTHDF2 and YTHDC2 are involved in regulating ferroptosis. While m^6^A regulators are involved in controlling ferroptosis, RNAs targeted by m^6^A in this process remain unclear [107–109].

### Tumor angiogenesis

Angiogenesis within the tumor microenvironment ensures sustained blood supply for tumor progression and emerging evidence indicates that hypoxia serves as a key driver of tumor angiogenesis in part through mediating m^6^A modification of mRNAs to promote mRNA stability [110]. In both human and mouse HCC, loss of YTHDF2 disrupts m^6^A-dependent mRNA decay of inflammation and angiogenesis-related mRNAs including IL-11 and SERPINE2 and the resulting increase in of IL-11 and SERPINE2 expression reshapes the HCC microenvironment by promoting inflammation and vascular remodeling [111]. Interestingly, hypoxia has been found to negatively regulate YTHDF2 expression, and treatment with PT2385, a HIF-2α inhibitor, restores YTHDF2 expression in HCC [111]. In CRC, IGF2BP3 promotes vasculature reconstruction through reading the m^6^A-modified mRNA encoding vascular endothelial growth factor (VEGF) promoting its stabilization and increasing its expression. This is in addition to the role of IGF2BP3 in sustaining cyclin D1 mRNA stability leading to cell cycle progression at the G1 to S phase transition to promote cancer cell proliferation [112]. These phenotypical outcomes further support an important role for m^6^A modification of RNA in modulating angiogenesis.

### Tissue invasion & metastasis

Local invasion and distant metastasis are the most important causes for cancer associated death in patients. The Epithelial-mesenchymal transition (EMT) is regarded as an initial step of this process and is regulated in part by m^6^A modification [112]. Snail, a key transcription factor involved in the EMT, is positively regulated by METTL3 and YTHDF1 through its m^6^A-modified mRNA coding sequence to promote translation efficiency of Snail [40]. Consistent with this, deletion of METTL3 suppresses in vitro EMT-related phenotypes in HCC cells including cell migration and invasion [40]. Similarly, METTL3 was identified as a crucial inducer of metastasis for ESCA in an in vivo CRISPR screen. Mechanistically, METTL3 serves as the m^6^A transferase of EGR1 mRNA, leading to its stabilization and activation of the EGR1/Snail regulatory loop, resulting in cancer cell metastasis [113]. Furthermore, in GC, higher expression of METTL3 promotes cancer cell metastasis in vivo and is predictive of poor prognosis in GC patients, which is possibly through the m^6^A modified stabilization of the zinc finger MYM-type containing 1 (ZMYM1) mRNA. ZMYM1 recruits the CtBP/LSD1/CoREST complex to the promoter of CDH1 (E-cadherin gene), thus inducing the EMT program [114]. METTL14, also inhibits CRC migration and invasion during the EMT, partially through regulating SOX4 in an m^6^A-YTHDF2-degradation dependent manner [115]. Also in the CRC, by utilizing the AOM/DSS combined YTHDF1 transgenic mice model, YTHDF1 was shown to promote tumorigenesis and metastasis through targeting m^6^A-ARHGEF2, which activates RhoA signaling and promotes the induction of stress fibers and focal adhesions [116]. Another component of the MTC, KIAA1429 (termed VIRMA), also has higher expression in HCC than normal liver tissue and enhances migration and invasion of HCC through modulating the ID2 mRNA or GATA3 pre-mRNA in an m^6^A-dependent manner [114, 117].

**Fig. 3:**
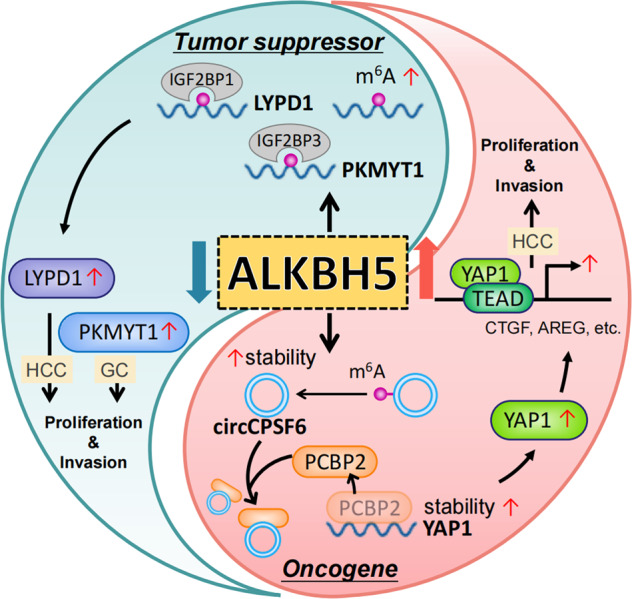
The “yin and yang”effect of ALKBH5 in GI cancer development. The oncogenic ALKBH5 demethylates m^6^A modified circCPSF6 to promote expression of YAP1 by blunting PCBP2 binding to YAP1 mRNA, to facilitate HCC development. The tumor suppressor role of ALKBH5 is revealed by its loss of expression in HCC and GC, which facilitate the m^6^A modification of LYPD1 and PKMYT1 contributing to mRNA stabilization and tumor proliferation and invasion in IGF2BP-dependent manner.

Increasing evidence suggests that m^6^A regulatory factors may play a role in malignant processes in a context-dependent manner, while ALKBH5 is one of the most prevalent GI cancer metastatic elements (Fig. 3). ALKBH5 promotes the invasion and metastasis of GC by demethylating lncRNA nuclear paraspeckle assembly transcript 1 (NEAT1) [117]. Similarly, in HCC, ALKBH5 could demethylate and stabilize the expression of circular RNA circCPSF6, which stabilizes YAP1 mRNA by competitively binding to the PCBP2, resulting in HCC metastasis [118]. Contrary to these findings, lower expression of ALKBH5 is correlated with the distal metastasis and lymph nodes metastasis of GC patients [119]. Loss of function of ALKBH5 cooperates with IGF2BP3 to stabilize PKMYT1, thus promoting the invasiveness of GC cells both in vivo and in vitro. Similarly, expression of ALKBH5 is also decreased in CRC cells, which promotes invasion and migration of CRC cells [120]. Also, decreased level of m^6^A modification arising from ALKBH5 induces the Wnt signaling activation and metastasis of both PDAC cells and patient-derived xenograft (PDX) models through decreasing the expression of Wnt inhibitory factor 1 (WIF-1) [121]. These seemingly contradictory phenomena should be assessed in future in vivo studies such like the conditional knockout mice to define the role of ALKBH5 in metastasis of GC and other cancers types.

### Metabolic reprograming

Unlike normal cells, cancer cells meet their high energy demands via reprogramming cell metabolism including aerobic glycolysis and lipid homeostasis. During the process of glycolysis, the conversion of glucose to glucose-6-phosphate is controlled by the enzyme hexokinase (HK) and GLUT1, both of which are highly pertinent to tumorigenesis [122–124]. Not surprisingly, the role of m^6^A in regulation of HK and GLUT1 would influence cancer cell metabolism. To this end, METTL3 targets HK2 and GLUT1 mRNA for m^6^A leading to their stabilization in IGF2BP2- and IGF2BP2/3-dependent mechanisms in CRC [125]. This phenomenon was also demonstrated using the patient-derived CRC organoids and METTL3 knockout mouse models [126]. Similarly, overexpression of METTL3 promotes glycolysis through the METTL3/HDGF/GLUT4/ENO2 axis in GC [120], which promotes liver metastasis of GC cells. Furthermore, METTL3 increases expression of LINC00958, a lipogenesis-related lncRNA, through m^6^A-mediated RNA stabilization. The increased LINC00958 in turn upregulates HDGF expression via miR-3619-5p, facilitating HCC lipogenesis, and tumor progression [101]. These studies suggest the METTL3-HDGF axis plays an important role in cancer glycolysis and lipid metabolism. In addition, METTL3 promotes GLUT1 translation in an m^6^A-dependent manner, which subsequently elevates glucose uptake and lactate production, leading to the activation of mTORC1 signaling and CRC development [104]. In HCC, ALODA, a key enzyme in glycolysis, was shown to be regulated by FTO mediated-m^6^A in a YTHDF2-dependent manner in HCC [127]. SIRT6, another protein serving as a tumor suppressor via inhibiting aerobic glycolysis in diethylnitrosamine-induced liver tumorigenesis, is also regulated by the METTL14-m^6^A-USP48 axis [128]. Another key mechanism controlling cancer metabolism are amino acid levels. In addition to cancer cells synthesizing their own amino acid pools, they often require additional, external amino acid sources, raising the possibility of amino acid deprivation therapy for cancer treatment [129]. Though there is no direct evidence to elucidate the mechanism for m^6^A-regulated amino acid metabolism, a series of vital signature pathways such as mTOR have been reported to be tightly controlled by m^6^A, thus providing a novel avenue for cancer therapy by targeting a m^6^A-amino acid axis [130].

### Cancer immunoevasion

With significant advances in cancer immunotherapy, such as immune checkpoint therapy (ICT) and CAR-T, the tumor immune microenvironment (TIME), which is infiltrated with many types of innate or adaptive immune cells, influences the efficacy of these novel therapeutic strategies. As might be anticipated, m^6^A RNA modification participates in regulating the TIME of GI cancers, both through the tumor cells as well as the various immune cells. Our understanding of the m^6^A-related cancer immunity rapidly increased over the last decade, and we summarize this progress in GI cancer here (Table 2).

**Table 2:** m^6^A-mediated regulation of diverse immune cells in the tumor microenvironment.

Regulation of lymphoid cell functions						
Cell types	Regulators	m^6^A substrate	Cancer types	Mechanism	Phenotype	Reference(PMID)
T cells	METTL3 METTL14	Stat1, Irf1	CRC	Depletion of METTL3 or METTL14 inhibit the m^6^A level but stabilizes Stat1 and Irf1 mRNAs, hence activating IFN-γ-Stat1-Irf1 signaling.	Depletion of METTL3 and METTL14 enhances response to anti-PD-1 treatment in pMMR-MSI-L CRC and melanoma; METTL3 or METTL14 deficient tumors increased cytotoxic tumor-infiltrating CD8^+^ T cells and elevated secretion of IFN-γ, Cxcl9, and Cxcl10 in tumor microenvironment in vivo.	32964498
T cells	ALKBH5	Mct4, Slc16a3	CRC	ALKBH5 regulated Mct4 expression by decreasing its m^6^A levels and RNA stability.	Suppressing Tregs and myeloid-derived suppressor cell accumulations; ALKBH5 knockout in tumor cells enhances efficacy of immunotherapy and increased mouse survival.	32747553
T cells	YTHDF1	unknown	GC	Depletion of YTHDF1 promotes IFN-γ receptor 1 expression and enhances IFN-γ response, promoting expression of major histocompatibility complex class I (MHC I).	Promotes self-presentation of the immunogenic tumor cells to stimulate a strong cytotoxic T lymphocytes responses.	36484103
T cells	YTHDF1	PD-L1, VISTA	CRC	YTHDF1 regulates PD-L1 and VISTA expression in an m^6^A dependent manner.	YTHDF1 depletion inhibited tumor growth by enhancing the infiltration of CD8^+^ T cells and synergized with PD-1 blockade for cancer treatment.	35803704
T cells and NK cell	IGF2BP1	c-MYC, KRAS	HCC	CuB (Cucurbitacine B) promoted IGF2BP1-dependent target mRNA instability.	Induces tumor cell apoptosis, and recruits various immune cells (CD4^+^ and CD8^+^ T cells, CD56^+^ NK cells, and F4/80^+^ macrophages).	36032766
NK cells	METTL3	SHP2	CRC	METLL3 depletion attenuates the m^6^A modification and stability of SHP-2, thus activating AKT and MAPK signaling pathway.	Inhibits NK cell infiltration and function, leading to tumor growth and shortened survival in mice.	34535671
**Regulation of myeloid cell functions**						
**Cell types**	**Regulators**	**m**^**6**^**A substrate**	**Cancer types**	**Mechanism**	**Phenotype**	**Reference**
TIMs (including TAMs, TANs, MDSCs)	METTL3	Jak1	CRC	METTL3 mediated m^6^A modification on Jak1 mRNA in TIMs, the m^6^A-YTHDF1 axis enhanced JAK1 protein translation efficiency and subsequent phosphorylation of STAT3.	METTL3/JAK1/STAT3 axis strengthens immunesuppressive functions of myeloid cells.	35320754
MDSCs	METTL3	BHLHE41	CRC	METTL3 promoted BHLHE41 expression in an m^6^A-dependent manner.	METLL3 enhances MDSC migration in CRC.	35700773
MDSCs	YTHDF1	p65	CRC	YTHDF1 activates CXCL1-CXCR2 axis by promoting m6A-p65 translation.	Promoting MDSC migration and antagonizing functional CD8^+^ T cells it the TIME.	36717220
Macrophages	METTL14	Ebi3	CRC	Loss of METTL14 in C1q+ macrophages promote the accumulation of Ebi3 mRNA in an m^6^A dependent manner.	METTL14 deficiency in macrophages impairs the antitumor response and CD8^+^ T cell dysfunction, contributing to tumor growth.	34019807
Macrophages and MDSCs	ALKBH5	PD-L1	ICC	Loss of ALKBH5 enhances the m^6^A modification on the 3’ UTR of PD-L1 mRNA accelerating its degradation in a YTHDF2-dependent manner.	ALKBH5 promotes the expression of PD-L1 on monocyte/macrophage cells decreasing the infiltration of MDSC-like cells to the TIME.	34301762
DCs	YTHDF1	CTSA, CTSB, CTSD, CTSH	CRC	Transcripts encoding lysosomal proteases are marked by m6A and recognized by YTHDF1, thus increasing the translation of lysosomal cathepsins in dendritic cells.	YTHDF1 depletion inhibits cathepsins to enhance cross-presentation of wild-type dendritic cells. Enhanced antigen-specific CD8^+^ T cell anti-tumor response and PD-L1 blockade efficacy.	30728504
DCs	YTHDF1	IFN-γ1	GC	Depletion of YTHDF1 amplifies DC-mediated anti-tumor immune response including CD4^+^ and CD8^+^ T cells infiltration with increased IFN-γ secretion.	Promoting GC by inducing cell proliferation and repression of DC-mediated antitumor immune response including CD4^+^ and CD8^+^ T cells infiltration with increased IFN-γ secretion	35193930

#### Regulation of lymphoid immune cell functions

Lymphoid cells, especially T cells, are the fundamental effectors in adaptive immune system, serving as the major player in cancer immune phenotype. Conditional knockout of METLL3 impairs the homeostasis of T cell subsets including CD4^+^ subset, Tregs, and T follicular helper (Tfh) cells [12, 131, 132]. In the TIME, depletion of METTL3, or METTL14 depletion within CRC tumors, increases cytotoxic tumor-infiltrating CD8^+^ T cells and secretion of cytokines such as IFN-γ, CXCL9 and CXCL10, hence sensitizing tumors to PD-1 antibody treatment. Mechanically, METTL3 or METLL14 deficiency inhibits m^6^A levels, but stabilizes Stat1 and Irf1 mRNAs, hence activating IFN-γ-Stat1-Irf1 signaling [133]. Interestingly, a CRISPR screen identified the demethylase ALKBH5 as also assist CRC cell evasion from immunological surveillance realized by modulating the suppressive Treg and myeloid-derived suppressor cell accumulation. Thus, ALKBH5 knockout in CRC cells enhance efficacy of immunotherapy and pro-longed mouse survival time.

Similarly in CRC models, higher expression of YTHDF1 recognizes m^6^A modified PD-L1 and VISTA to enhance their stability, thus serving as the ideal target to promote the infiltration of CD8^+^ T cells, when synergizing with PD-1 blockade [134]. Considering its the potential effects on immune evasion, YTHDF1 siRNA is packaged in engineered small extracellular vesicles (sEVs) for GC treatment, producing anti-cancer efficiency by achieving self-presentation of the immunogenic tumor cells to stimulate robust cytotoxic T lymphocytes responses [135]. Similarily, after confirmation of IGF2BP1 as a crucial regulator of T cell composition in the TIME of HCC, an IGF2BP1 small molecule inhibitor exhibited the ability to recruit not only CD4^+^ and CD8^+^ T cells, but also CD56^+^ NK cells and F4/80^+^ macrophage, into the TIME [136].

On another note, while studies are lacking assessing m^6^A regulated B cell dysregulation in TIME, B cells in the germinal center of a non-tumor model, are tightly controlled by METTL3 and METTL14, indicating their potential application in potential B cell targeted immunotherapy in the future [137, 138].

Besides T and B cells, NK cells are also lymphatic derived components of the TIME, and have the capacity to directly recognized and kill cancer cells. METTL3 also participates in the maturation of NK cells. In the TIME, METTL3 expression is inhibited in infiltrating NK cells by tumor derived TGF-β. Attenuated METTL3 weakens the activation of AKT, mTOR, and ERK signals in an m^6^A-dependent manner, resulting in blockage of the IL-15 response and maturation of NK cells [139]. Knockout of METTL3 in NK cells inhibits their infiltration and function, leading to CRC development and shortened survival in mice model. The detailed mechanism appears to be that loss of METLL3 destabilizes SHP-2 leading to insensitivity to IL-15 and inhibiting AKT and MAPK signaling pathways [139].

#### Regulation of myeloid immune cell functions

Myeloid derived immune cells including MDSC (myeloid-derived suppressor cell), monocytes (such as dendritic cell (DC), macrophages, and granulocytes (such as Neutrophiles) are also components of the TIME. Knockout of METTL3 in tumor-infiltrating myeloid cells (TIMs) including tumor-associated macrophages (TAM), tumor-associated neutrophiles (TAN), and MDSCs disrupts the immunosuppressive functions of myeloid cells in the CRC model, which is mediated by a METTL3-JAL1-STAT3 signal cascade [140]. MDSCs are a heterogeneous group of immature myeloid cells that participate in the immunosuppressive microenvironment in GI cancer [141]. Depletion of METTL3 in the spontaneous CRC mouse model leads to reduced MDSCs but increased CD4^+^ and CD8^+^ T cell infiltrates. High expression of METTL3 enables CRCs to recruit MDSCs by catalyzing m^6^A-BHLHE41 to enhance the secretion of CXCL1, thus contributing to T cell growth inhibition and CRC development [142]. In a recent report, intestine-specific YTHDF1 knock-in promotes MDSC migration, antagonizing functional CD8^+^ T cells in the TIME enhancing CRC growth. In this case, YTHDF1 activates the CXCL1-CXCR2 axis to promote m^6^A-p65 translation [143].

In macrophages, METTL3 knockout in myeloid cells facilitates tumor growth and lung metastasis, partially controlled by TAM augmentation and regulatory T cell infiltration into the tumor microenvironment [144]. Along these same lines, depletion of METTL14 in TAMs drives CD8^+^ T cell differentiation along a dysfunctional trajectory, impairing CD8^+^ T cells to eliminate tumors [145]. Besides, METTL14 cooperated with YTHDF2 to limit the expression of the Epstein-Barr virus-induced gene 3 (Ebi3) protein, a T cell state regulator. By utilizing single cell sequencing, METTL14 deficiency in a subset of TAMs distiquished by the marker C1q^+^, impairs the antitumor response by driving CD8^+^ T cell dysfunction, resulting in CRC tumor growth [146]. In addition to these writers, the eraser ALKBH5 but not FTO is also involved in macrophage related phenotypes. Loss of ALKBH5 enhances the m^6^A modification on the 3’ UTR region of PD-L1 mRNA to promote its degradation in a YTHDF2-dependent manner, thus decreasing the expression of PD-L1 on monocyte/macrophage cells and increasing the infiltration of MDSC-like cells in intrahepatic cholangiocarcinoma (ICC). ICC patients with lower expression of ALKBH5 show greater sensitivity to to anti-PD-1 immunotherapy [147]. Additionally, YTHDF2 is involved in regulating tumor-associated macrophages and Tregs in HCC [148, 149].

DCs, the main antigen-presenting cells (APC) serving as the bridge between the innate or adaptive immunity, are also tightly regulated by the m^6^A system in the TIME. For instance, METTL3 promotes dendritic cell (DC) maturation and function via enhancing translation of CD40, CD80, and the TLR4 signaling adaptor Tirap mRNAs [150]. YTHDF2 also upregulates the DC-based immune response by degrading m^6^A-modified lnc-Dpf3 [151]. In CRC, YTHDF1 promotes m^6^A-transcripts encoding lysosomal proteases, thus increasing the translation of lysosomal cathepsins in dendritic cells. YTHDF1 depletion causes inhibition of cathepsins that markedly enhance cross-presentation of wild-type dendritic cells, and enhanced antigen-specific CD8^+^ T cell response and increased PD-L1 blockade efficacy [152]. Similar effects mediated by YTHDF1 were also shown in GC. Depletion of YTHDF1 amplifies DC-mediated anti-tumor immune response including CD4^+^ and CD8^+^ T cells infiltration with increased IFN-γ secretion [153]. Neutrophiles are also regulated by m^6^A regulators METTL3 and ALKBH5, but no direct evidence indicating their regulation in the TIME has been described and thus requires further research [154, 155].

With the development of cancer immune therapy, myriad of multi-omics data including bulk or single cell RNA sequencing, ATAC, and m^6^A related sequencing, has been deposited in public repositories which has given rise to a series of bioinformatic based project to assist in development of patient targeted therapy strategies based on the status of varous m^6^A regulators. For instance, evaluation of m^6^A modification patterns within individual tumors could predict tumor inflammation stage and prognosis in GC [156]. Tumors with low m^6^A scores have a higher 5-year survival rate due to increased mutation burden and activation of anti-tumor immunity. In contrast, lack of effective immune infiltration is observed in tumor subtypes with higher m^6^A scores and is associated with poorer survival. Similar studies have also been conducted in PDAC, CRC, and HCC [157–160]. These studies reveal the essential role of m^6^A modification in tumor immunity. Considering the complexity of the TIME, future studies should focus on the heterogeneity as well as the potential communication of the m^6^A modification between different components in the TIME.

### Cancer stem cell property

Cancer stem cells (CSCs) are regarded as “seed cells” contributing to various types of malignancies in GI cancer [161–163]. Dysregulation of m^6^A regulatory mechanisms may impact stemness traits of GI cancer by interfering with CSC-related pathways. For example, METTL3 induced m^6^A-modified CBX8 promotes expression of LGR5, a well-established marker of gastric and colorectal cancer stem cells, through recruiting Pol II and KMT2b transcriptional complexes to maintain stemness of CRC CSCs and promoting drug resistance [111]. Similarly, METTL3 methylates SOX2 transcripts to prevent their degradation through IGF2BP2 binding to the CDS region of the SOX2 mRNA in CRC cells. Consistent with this, METTL3 depletion drastically inhibits CSC self-renewal, stem cell frequency, and migration, thereby suppressing CRC tumorigenesis and metastasis [164]. IGF2BP2 has also been reported to promote proliferation and stemness-like properties of PC cells by binding and stabilizing m^6^A-modified DANCR, a lncRNA, involved in preservation of CSC stemness [165, 166]. A positive correlation between YTHDF1 levels and stemness signatures in CRC was recently identified via gene set enrichment analysis [167]. YTHDF1 deficiency results in suppression of colonosphere self-renewal to promote differentiation by repressing frizzled class receptor 9 and Wnt family member 6 [167]. However, the interplay between YTHDF1 and FZD9/WNT6 needs further exploration. Another report demonstrated that the liver progenitor specific gene RALYL is associated with unfavorable prognosis and poor differentiation in HCC patients. Mechanically, RALYL cooperated with FTO to demethylate and stabilize TGF-β2 transcripts, resulting in enhanced TGF-β signaling and subsequent PI3K/AKT activation, which contributes to enhanced HCC CSC stemness [168]. These studies may offer new therapeutic approaches for GI cancers by targeting m^6^A regulators in CSCs.

### New dimensions

Most recently, the traditional hallmarks of cancer have been updated and novel characteristics such as phenotypic plasticity, disrupted differentiation, nonmutational reprogramming, and polymorphic microbiomes have gained consideration [169]. Emerging evidence supports the relationships between various aspects of these new cancer hallmarks and m^6^A RNA modifications. For instance, evading terminal differentiation, otherwise known as unlocking phenotypic plasticity, may be regulated by METTL3 and METTL14 [170]. Sox2 is a stemness-related TF that regulates the conversion between differentiation and dedifferentiation. In glioblastoma, METTL3 is indispensable for sustaining Sox2 mRNA stabilization which fine-tunes stem cell characteristics and dedifferentiation [170]. Conversely, another m^6^A writer, METTL14, together with the IGF2BP reader proteins, mediate the hepatocyte nuclear factor HNF3γ, which promotes HCC cell differentiation and inhibits the dedifferentiation process [102]. Although there is currently no direct evidence of a regulatory role for m^6^A in cancer cell transdifferentiation, a series of transdifferentiation-related regulators or signaling pathways such as p53, H19, and mTOR signaling are broadly mediated by m^6^A regulators, which needs to be further explored to better understand tumor phenotypic plasticity [168, 171, 172].

In addition to impacts of tumor or stromal cells on tumorigenesis, polymorphic variability in the microbiomes of rodent models and patients are now considered to be an important modulator for cancer progression, especially in GI cancers [173]. A role for m^6^A modification in microbiota-host-interaction in the intestine and liver, which can interfere with the host nutrient metabolism, provides another scenario for m^6^A modification [174]. For instance, *Fusobacterium nucleatum* (*F. nucleatum*), a CRC enriched intestinal bacteria, could activate YAP signaling which inhibits transcription of forkhead box D3 (FOXD3), an essential TF regulating METTL3 expression. Downregulation of METTL3 promotes CRC metastasis by decreasing m^6^A modification of kinesin family member 26B (KIF26B) [175]. This finding gives rise to a detailed mechanism regarding how the gut microbiome may coordinate cancer cell metastasis, clarifying the concept that the microbiota in organisms may affect epigenome plasticity, such as m^6^A modification, to control the progression of pathophysiologic processes. Another interesting topic has arisen through a series of studies indicating the existence of microbiota residing inside the tumor cell, even in glioma, a generally considered closed and germfree carcinoma [171, 176]. It will be interesting whether there is a genome or epigenome connection between tumor cells and their resident microbiota.

Cellular senescence has long been considered a complementary mechanism to programmed cell death. While initially a tumor suppressive mechanism to halt tumorigenesis induced by such factors as oncogene activation, it also plays a cell-nonautonomous role in promoting tumor progression through the senescence-associated secretory phenotype (SASP) [177, 178]. METTL3 was thought to alleviate mesenchymal stem cell senescence in a m^6^A-mediated manner, which is a potential strategy for cellular rejuvenation [179]. On the contrary, METTL3 was also observed to promote the SASP by decreasing m^6^A modification of ATG7 mRNA, accelerating cartilage destruction in osteoarthritis progression [180]. Two controversial results hint that the function of this m^6^A mediator in regulating cell senescence needs further exploration. In tumor models, METTL3 and METTL14 were reported to redistribute and localize to the promoters or enhancers of SASP genes during the senescence process. This relocalization appears indispensable for the SASP, resulting in tumorigenesis and immune-surveillance functions of senescent cells in vivo [181].

## Targeting RNA m^6^A regulatory pathways for anti-cancer therapeutics

### Targeting FTO and ALKBH5

**Fig. 4:**
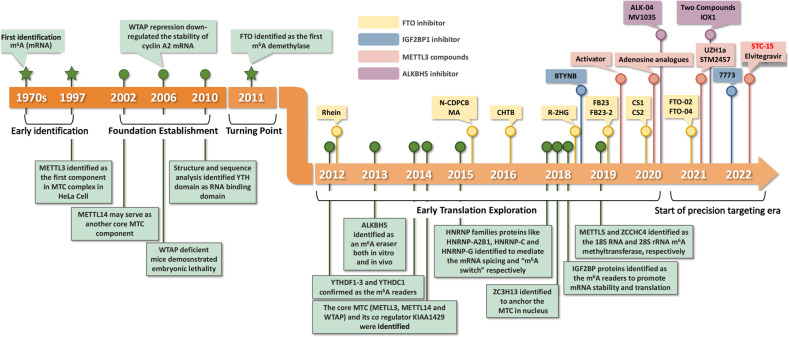
A brief timeline of m^6^A targeted therapy. Since its identification in the 1970s, m^6^A has been shown to be one of the most abundant internal mRNA modifications, that remained poorly understood until the discovery of the first m^6^A methyltransferase METTL3. In the next decade, a series of core components of m^6^A methyltransferase complex (MTC) including METTL14 and WTAP were identified to establish the basic theory of m^6^A system. The turning point for this field occurred in 2011 when FTO, the first m^6^A demethylase, was identified which gave credence to the reversibility of m^6^A. From 2012 to 2020, m^6^A related research advanced rapidly especially in the identification of m^6^A regulators as well as their roles in the hallmarks of cancer. In this period, the canonical m^6^A system coordinated by writer, eraser, and reader proteins were established. In addition, targeting compounds were developed and demonstrated to have anti-cancer effects especially for the reader FTO, revealing its therapeutic potential. In the current era of m^6^A targeted therapeutic development, compounds are in preclinical and early clinical development, include the METLL3 inhibitor STM2457 and its derivative STC-15, which has been submitted to the FDA for a Phase 1 clinical trial in cancer patients.

Given a critical role for m^6^A in promoting the hallmarks of GI cancers, m^6^A regulators may serve as promising molecular targets for cancer therapy (Fig. 4). Among these regulators, FTO is one of the most well-established targets. To date, several FTO inhibitors have been discovered by cell-based small-molecule compound library screens, natural product testing, or targeted chemical synthesis. The first FTO inhibitors like rhein have been identified through structure-based virtual screening [182]. Rhein is capable of repressing m^6^A demethylation in cells by competitively binding the FTO catalytic domain and disrupting FTO from binding m^6^A RNAs [182]. Additionally, CHTB and N-CDPCB have also been identified as FTO inhibitors [183, 184]. The crystal structures of N-CDPCB or CHTB complexed with FTO reveal that N-CDPCB is sandwiched between an antiparallel β-sheet and the L1 loop of FTO, whereas CHTB binds to the surface area of the FTO active site. Meanwhile, meclofenamic acid (MA), a nonsteroidal anti-inflammatory agent, was identified to specifically inhibit FTO by competitively binding to FTO sites of m^6^A-modified oncogenic mRNAs, thus effectively suppressing the cancer cell proliferation [185]. Similarly, FB23 and FB23-2, two derivatives of MA, dramatically suppress proliferation and promote differentiation and apoptosis of acute myelocytic leukemia cells by inhibiting FTO m^6^A demethylase activity [186]. Interestingly, the in vivo effects of R-2-hydroxyglutarate (R-2HG), a FTO inhibitor, have been tested by systemic administration in mice [187]. R-2HG inhibits FTO demethylase activity to increase overall m^6^A levels, which destabilizes MYC and CEBPA transcripts, resulting in anti-tumor effects in vitro and in vivo [187]. Moreover, CS1 and CS2, two potent small molecule inhibitors of FTO, exert effective anti-tumor effects by suppressing immune checkpoint gene expression, especially LILRB4, in an m^6^A-dependent manner, thus sensitizing cancer cells to T cell cytotoxicity in leukemia and PC models [188]. This finding suggests translational applicability of m^6^A-based targets in cancer immunotherapy. In addition, FTO depletion contributes to increased sensitivity to interferon gamma (IFNγ) and anti-PD-1 by enhancing m^6^A-modified PD-1, CXCR4, and SOX10 degradation in melanoma cells, implying a clinical benefit of FTO inhibition combined with anti-PD-1 blockade in overcoming the resistance in cancer immune checkpoint therapy (ICT) [189].

However, these FTO inhibitors appear unsuitable for clinical use due to either poor target selectivity or pharmacokinetics. Recently, two novel FTO inhibitors, FTO-02 and FTO-04, were identified by fluorescence enzymatic inhibition assays, which show specificity toward FTO over ALKBH5. Importantly, FTO-04 prevented tumorsphere formation in patient-derived glioblastoma stem cells (GSCs) without inhibiting the growth of healthy neural stem cell-derived neurospheres [190]. Recently, FTO was shown to regulate chromatin plasticity and accessibility through removing m^6^A from carRNAs [35], expanding the regulatory role of m^6^A more broadly to the genome level, which needs to be carefully considered for the future exploration of the m^6^A related compound selection and target scanning, as well as clinical application.

The first generation of ALKBH5 inhibitor, ALK-04, was identified through in silico screening using X-ray crystal structures of ALKBH5 [179]. Combination of ALK-04 and PD-1 antagonist display synergetic anti-cancer effects in a syngeneic B16-based mouse model [179]. Recently, a series of novel compounds against the ALKBH5 catalytic activity were identified utilizing the FIMM compound library. One compound, 4-{[(furan-2-yl)methyl]amino}-1,2-diazinane-3,6-dione [6], was further verified to inhibit proliferation in leukemia cell lines [191]. Since ALKBH5 belongs to 2-oxoglutarate (2-OG)–dependent enzyme family, broad-spectrum 2-OG oxygenase inhibitors such as IOX1 and MV1035 also display inhibitory effect against ALKBH5 activity [192–195].

### Targeting other m^6^A regulators

Although a series of inhibitors against eraser proteins show potential anti-cancer effects, METTL3, the core factor of the MTC, may be the ideal target in the m^6^A network. Two adenosine analogs have been reported as potential METTL3 inhibitors based on high-throughput screening and X-ray crystallography. But the effect of both compounds on METTL3 activity has yet to be tested in vivo [196]. Subsequently, a small molecule named UZH1a was discovered and its binding affinity with METTL3 was confirmed whereas its enantiomer UZH1b by was shown not to bind [197]. UZH1a inhibits m^6^A modification in a wide range of cell lines. More importantly, it shows high selectivity to METTL3 compared to other methyltransferases such as METTL5 or ZCCHC4 [197]. More recently, Elvitegravir, an FDA-approved integrase inhibitor for the treatment of HIV, was identified as a novel METTL3 inhibitor and inhibits ESCA metastasis instead of limiting proliferation in animal models [113].

The first METTL3 catalytic inhibitor to be phenotypically tested, STM2457, was developed in 2021 after high-throughput screening of 250,000 diverse drug-like compounds [198]. STM2457 displays high affinity for the SAM-binding site of METTL3 compared to other RNA, DNA or protein methyltransferases, thus reducing global m^6^A levels and mRNA translation efficiency. Treatment of tumors with STM2457 suppresses growth of acute myeloid leukemia (AML) as well as an increase in differentiation and apoptosis. Furthermore, inhibition of METTL3 by STM2457 in vivo leads to impaired engraftment and prolonged survival in various mouse models of AML. In CRC, STM2457 combined with PD-1 antibody demonstrates a synergistic effect, characterized by enhanced suppression of tumor volume and increased CD4^+^ or CD8^+^ T cells infiltration [142]. Interestingly, STM2457 also restrict HCoV-OC43 and SARS-CoV-2 replication through restraining the m^6^A modification on β-coronavirus RNA [199]. It is worth noting that STC-15, a more advanced compound compared to STM2457, has move into the first stage of clinical trial (NCT05584111) to systematically assess safety and tolerability, pharmacokinetics, pharmacodynamics and clinical activity of STC-15 in adult subjects with advanced malignancies this year. This first-in-human study may provide the precious results to guide the future work in m^6^A related anti-cancer therapy.

METTL3 activators have also been developed to open new possibilities in m^6^A-targeted pharmacotherapeutics in future clinical applications, which may prove useful as the function of m^6^A mediators in tumorigenesis is often viewed as context-dependent [200].

In addition, utilizing high-throughput fluorescence anisotropy/polarization microplate assay (FAMA), BTYNB was identified from more than 160,000 molecules to block the interaction of IGF2BP1 with its substrate RNAs [201]. Further studies have demonstrated that BTYNB treatment interferes with E2F-driven gene expression regulated by IGF2BP1 in a m^6^A-dependent manner, resulting in cancer cell cycle arrest [202]. More recently, two novel inhibitors, cucurbitacin B (CuB) and 7773, were identified that block the KH1-2 and KH3-4 domains of IGF2BP1, respectively. C-myc is the likley target for CuB while 7773 reduces KRAS mRNA abundance and additional IGF2BP1 downstream targets [203]{Liu, 2022 #209}. Interestingly, phenotypic alterations and related molecular targets seem to largely differ between BTYNB and 7773, which is partially explained by the six putative RNA binding domains of IGF2BP1. This feature may inspire development of more precise inhibitors against the m^6^A readers due to the context-dependent epigenetic traits of cancers.

## Conclusions and future directions

Since the m^6^A modification serves as a central hub in RNA metabolism, its pathophysiological roles have been increasingly linked to various human cancers. Notably, the context-dependent roles of m^6^A regulators in different cancer types warrant further investigation, which will be pivotal for rational targeting of m^6^A modification as cancer therapeutics. For example, METTL14 is decreased in CRC and acts as a tumor suppressor to inhibit cancer cell proliferation, migration, and invasion, while its expression is increased in pancreatic cancer, serving as an oncogene to sustain the tumor growth and metastasis [204–206]. One possible explanation is that the context-dependent expression or regulatory mechanism of m^6^A modification is dictated by specific oncogenic insults in cancers. For instance, hypoxia stimulates HIF-1α- and HIF-2α-dependent expression of ALKBH5, which demethylates NANOG mRNA and increased the percentage of breast CSCs in the tumor microenvironment [207].

Moreover, m^6^A modification can be dynamically regulated by various tumor microenvironment signals, while this field is just beginning to be explored. Recently, lactate accumulation, a tumor micorenvironment condition of solid tumors, also directly regulates METTL3 expression, which depends on H3K18 lactylation. Interestingly, the METTL3 protein itself is targeted for lactylation in its zinc-finger domain, which is crucial for m^6^A-RNA capture [140]. METTL3 was shown to be regulated by DNA damage and ERK signaling pathways. More specifically, METTL3 is phosphorylated at serine-43 by ATM and then translocates to double-strand DNA break sites to increase RNA:DNA hybrids that accelerate homologous recombination [208]. Meanwhile, activation of ERK phosphorylates METTL3 and stabilizes its expression by increasing USP5-mediated deubiquitylation, resulting in ERK-activated cancer cell tumorigenesis [209]. These studies suggest that METTL3 inhibition may be a promising strategy for cancer treatment either alone or combined with other approaches such as kinase inhibitors or chemo-radiotherapy. More recently, two studies reported that WTAP is involved in mTOR signaling, which supports the potential application of mTOR inhibitors for m^6^A-driven tumorigenesis [144, 210]. With the expansion of the transgenic models with greater pathophysiologic and clinical representation, these issues could be addressed in a more rational way. Besides GI cancer models summarized in this review, CRC, HCC and chemical induced mouse models account for the majority (Table 3).

**Table 3:** The roles of m^6^A regulators in GI cancers as revealed using genetically engineered mouse models.

m^6^A regulators in mouse models of GI cancers					
Regulator	Tumor type	Gene background	Cre	Tumor phenotype	Reference (PMID)
Genetic editing in cancer cells					
METTL3	CRC	*Apc*^*min/+*^*;Mettl3*^*+/−*^	N/A	Tumor suppression	35700773
METTL3	CRC	*Apc*^*min/+*^*;Mettl3*^*+/−*^	N/A	Tumor suppression Glucose uptake inhibition	33217448
FTO	HCC	*Fto*^*fl/fl*^	Alfp-Cre (hepatocyte-specific)	Tumor promotion	32956847
YTHDF1	HCC	*Ythdf1*^*fl/fl*^	Alb-Cre (liver-specific)	Autophagy Suppression of growth and metastasis	33619246
YTHDF1	CRC	*Ythdf1*^*fl/fl*^	N/A	Suppression of proliferation and metastasis	34968454
YTHDF1	CRC	*Apc*^*Min/+*^*;Ythdf1*^*+/+*^	CDX2-cre (intestine specific)	Tumor promotion and suppression	36717220
Genetic editing in stroma cells					
METTL3	CRC	*Mettl3*^*fl/fl*^	LysM-Cre (myeloid cell specific)	Enhanced tumor growth	35320754
METTL3	CRC	*Mettl3*^*fl/fl*^	Ncrl-Cre (NK cell specific)	Enhanced tumor growth and metastasis	34535671
METTL3	CRC	*Mettl3*^*fl/fl*^	LysM-Cre (myeloid cell specific)	Tumor growth	34019807
METTL14		*Mettl14*^*fl/fl*^			
YTHDF2		*Ythdf2*^*fl/fl*^			
YTHDF2		*Ythdf2-*3×FLAG-GGS-Avi-GGS			

Another open question in this area is the underlying mechanisms regarding how the various m^6^A modulators specifically recognize their substrates, which remains poorly understood, though it is well documented that the m^6^A modification preferentially occurs on specific RNA sequences. Among these modulators, readers like IGF2BPs harbor multiple RNA binding domains, which may bind different m^6^A modified RNAs, providing a potential model for better understanding selectivity of IGF2BPs to substrates. In the same way, the target specificity of IGF2BPs inhibitors may be realized by blocking the different RNA binding domains. But how writers and erasers choose their substrates, or whether there are cofactors involved in this process remains largely unknown. With recent understanding of phase separation, LLPS seems to be the rational mechanism to explain this selective process, in the consideration that m^6^A factors are involved in the establishment of various PS compartments (especially the role of m^6^A readers YTHDFs and YTHDC1), which coordinates the organization of specific m^6^A regulatory complexes [86, 88–90]. However, further studies are necessary to determine whether there exist divergent phase-separated compartments matching the m^6^A-RNA and reader composition, which although may be more complex might offer greater flexibility in gene regulation. Therefore, unraveling the mechanism(s) behind substrate preferences of m^6^A regulators contributes to personalized treatment for cancer.However, an easily overlooked point is that these m^6^A regulators may function in an m^6^A-independent manner. For instance, cytoplastic METTL16 may directly interact with eukaryotic initiation factor 3a (eIF3a) and eIF3b to recruit the translation-initiation complex, resulting in translation induction and tumorigenesis [145]. Similarly, KIAA1429 has an m^6^A-independent role in promoting the destabilization of WEE1 mRNA in CRC cells [211] which results in CRC progression.

Given the universal regulatory effects of m^6^A, other cell types constituting the tumor environment also need to be considered to elucidate the effect of m^6^A modifications and their contribution to malignant phenotypes. Recent studies have begun to touch on this area, especially in the context of the tumor immune microenvironment as we discussed extensivey in this review. However, some limitations still remain. First with respect to research models, most of the models in use are based on CRC and HCC, which are comparably accessible to achieve both in gene-editing or chemical induction compared with GC, which is more difficult to study using mouse models. Second, other cell types such as vascular and stromal cells, microbiota, and even viruses in the tumor microenvironment also warrant further study. With future breakthrough discoveries in this research direction we expect m^6^A-targeted therapy (single or combined) make progress in target population screening, treatment method selection, therapeutic response prediction, and patient prognosis assessment.

Altogether, m^6^A as the most abundant RNA chemical modification, has substantial importance in tumorigenic behavior through regulating both transcriptional or post-transcriptional mechanisms. Studies surrounding m^6^A in cancer development is an advancing field for cancer research, which will guide the direction of the next-generation anti-cancer treatment strategies. The clinical value of targeting m^6^A regulators, or the modified substrates, is worth looking forward to, though there remains many unanswered questions.
